# Western University Protocol for Obstructive Sleep Apnea

**DOI:** 10.3390/jcm15062385

**Published:** 2026-03-20

**Authors:** Rehab Simsim, Brian Rotenberg

**Affiliations:** 1 Department of Surgery, Medical College, Princess Nourah bint Abdulrahman University, P.O. Box 84428, Riyadh 11671, Saudi Arabia; 2 Department of Otolaryngology—Head and Neck Surgery, Western University, London, ON N6A 3K7, Canada; brian.rotenberg@sjhc.london.on.ca

**Keywords:** obstructive sleep apnea, snoring, drug-induced sleep endoscopy, CPAP intolerance, surgical protocol, sleep disordered breathing

## Abstract

**Background/Objectives**: Obstructive sleep apnea (OSA) is a prevalent disorder in adults, characterized by recurrent upper airway obstruction during sleep, resulting in intermittent hypoxia, sympathetic activation, and sleep fragmentation. It is linked to significant cardiovascular, metabolic, neurocognitive, and psychosocial morbidity. There is increasing evidence that continuous positive airway pressure (CPAP) adherence remains suboptimal in many patients, and in those patients, surgery is often indicated. **Methods**: This protocol report presents an updated and protocol-driven surgical approach grounded in clinical evidence and experience, highlighting the role of drug-induced sleep endoscopy (DISE) and personalized multi-level interventions for adult patients with OSA. The integration of anatomical phenotyping and DISE-directed planning enables precise surgical targeting. The protocol emphasizes patient selection, individualized treatment based on obstruction patterns, and perioperative optimization. This surgical algorithm improves the success rates and long-term outcomes in patients who are intolerant of CPAP therapy. **Results**: A DISE-guided and multi-level surgical approach includes uvulopalatoplasty, septoplasty, tongue base reduction, palatoplasty, and maxillomandibular advancement (MMA). Preoperative assessments include BMI and the STOP-BANG and Epworth Sleepiness scales, while postoperative care emphasizes follow-up polysomnography and adjunctive therapies only when necessary. Regional experiences in Saudi Arabia and Canada underscore the importance of standardized evidence-based surgical care. **Conclusions**: The purpose of this article is to establish a clear protocol for managing patients diagnosed with OSA, drawing on a review of the existing literature and the insights of experienced surgeons in the field of sleep apnea, and to update current protocols with modern evidence.

## 1. Introduction

Obstructive sleep apnea (OSA) is the most prevalent type of sleep-disordered breathing, currently affecting an estimated 1 billion people across the globe. Out of the total number of people affected, approximately 936 million of them are adults between the ages of 30 to 69 years old, and the disease can be classified as ranging from mild to severe. The pathophysiology of OSA is attributed to an anatomical and a neuromuscular component, both of which cause the recurrent collapse of the pharyngeal airway. These are interspersed with episodes of hypoxia and fragmentation of the sleep cycle [1,2]. These global burden estimates are based on a literature-based analysis [3]. In parallel, obesity—one of the strongest modifiable risk factors for OSA—has more than doubled since 1990, according to the World Health Organization [4].

OSA is independently associated with an increased risk of hypertension, coronary artery disease, stroke, insulin resistance, type 2 diabetes, mood disorders, and cognitive impairment. It significantly contributes to all-cause and cardiovascular mortality. This disorder also imposes a substantial economic burden due to elevated healthcare utilization and lost productivity [1,5].

While CPAP remains the most prominent treatment, some patients with mild-to-moderate OSA, nasal obstructions, claustrophobia, and those with a higher BMI often have long-term adherence rates below 50%. Discomfort with masks, dislike of the pressure, and the lifestyle burden of the CPAP machine often lead patients to discontinue CPAP therapy within the first 1–2 years. Oral appliance therapy (mandibular advancement devices) is an evidence-based alternative—particularly for patients with mild-to-moderate OSA or those intolerant of CPAP—and should be considered alongside other non-surgical and surgical options [6,7,8]. For these reasons, patients are recommended alternative treatment options in the case of CPAP intolerance, which include upper airway surgery and, for patients with higher BMIs, bariatric surgery [2,9].

OSA management in Saudi Arabia faces challenges due to a lack of awareness, insufficient diagnosis, and the uneven availability of services. Although the condition is highly prevalent, surgical interventions are not frequently employed due to a lack of multidisciplinary teams and the absence of standardized treatment protocols. Hence, CPAP is the first-line therapy, but it fails in a large proportion of patients [10].

Canada’s public healthcare system has significant diagnostic delays, sometimes extending up to 16 months, and instances of the underdiagnosis of OSA [11,12]. For adult patients who cannot tolerate or do not want CPAP, surgical interventions such as UPPP, barbed pharyngoplasty, septoplasty, rhinoplasty, tongue base reduction, palatoplasty, and maxillomandibular advancement (MMA) are available [13,14]. MMA is particularly effective, achieving a reduction in AHI of over 80% and allowing independence from CPAP therapy, but it carries a higher patient burden and lower uptake [15,16].

Conversely, the historically predominant model of surgical treatment for OSA has been the Stanford (Powell–Riley) staged phase I/II protocol. Phase I is an approach that is site-directed, multi-level, and usually involves retropalatal surgery (traditionally UPPP with or without tonsillectomy), plus treatment of the retrolingual obstruction (genioglossus advancement with or without hyoid myotomy/suspension) and correction of clinically significant nasal obstruction when present; postoperative PSG is then performed to re-evaluate the results after healing. Phase II surgery, involving maxillomandibular advancement (MMA), is then offered to patients with enduring clinically significant OSA following phase I, and this procedure is aimed at enlarging and stabilizing the entire pharyngeal airway with previously reported high success rates in the right patients [17].

Of OSA’s many comorbidities, hypertension, coronary artery disease, and stroke stand out, as they pose the greatest individual threat to the affected. Moreover, these conditions are the primary contributors to rising rates of mortality attributed to cardiovascular illness. In addition to the direct medical costs associated with a growing OSA population, there are also great indirect costs attributed to lost work productivity. Continuous positive airway pressure (CPAP) therapies are most often implemented as a primary line of treatment for OSA, yet persistent adherence to these protocols is estimated to be below 50% for most treatment recipients. This paper seeks to formulate an evidence-based, surgical-intervention pathway for adults with OSA who are deemed CPAP-intolerant [1,9].

## 2. Objective of the Study

Describing the practical implementation of a prototype multi-level surgical protocol across a variety of health care settings is a primary objective of this document. The protocol will be tailored to adults with obstructive sleep apnea who cannot tolerate CPAP, incorporating the latest evidence, expert opinion, and current health care practices to formulate a standardized DISE-guided protocol.

### 2.1. Diagnostic Evaluation

#### 2.1.1. Polysomnography (PSG)

PSG remains the gold standard for diagnosing OSA. It provides a comprehensive evaluation of the sleep architecture, respiratory disturbances, oxygen disturbances, and oxygen desaturation, and it monitors brain waves, muscle activity, heart rate, and breathing during sleep [18].

There are two types of sleep studies. One is an overnight sleep study conducted in a sleep lab or at home; the second type is home sleep apnea testing (HSAT), which is considered a level III study. The latter is often better accepted by patients due to the improved reliability and validity of testing conducted in their own bed with their own pillow. This study uses a portable device that measures breathing patterns. A sleep study monitors sleep patterns, breathing, and other physiological activities, which helps to diagnose sleep disorders and guide the treatment. Diagnostic definitions and indications for polysomnography and home sleep apnea testing follow contemporary American Academy of Sleep Medicine guidance [18].

Using AHI alone may not correctly show a significant clinical improvement. This protocol uses the SLEEP-GOAL framework proposed by Pang and Rotenberg as the main outcome measure, which focuses on multidimensional patient-centered outcomes. In a multicenter randomized trial of 302 adults undergoing nose, palate, and/or tongue surgeries, the overall success measured using conventional Sher AHI methods was 66.2%, compared to SLEEP-GOAL, which found another group of responders who experienced considerable benefits in terms of their blood pressure, body mass index/weight, and hypoxemia load (reduced oxygen saturation below 90%), although not according to AHI standards. SLEEP-GOAL is more sensitive to clinical changes in areas that matter to patients. Practically, SLEEP-GOAL achieves success according to changes in key areas (blood pressure, BMI/weight, nocturnal hypoxemia, AHI) that are complemented by symptom measures; it is a more global measure of surgical success than AHI minimization [19].

#### 2.1.2. Drug-Induced Sleep Endoscopy (DISE)

DISE enables the dynamic assessment of upper airway obstruction under sedation, better simulating natural sleep conditions than examinations while awake. This technique is widely used to individualize surgical planning [19,20]. It is especially useful in identifying obstruction patterns undetectable on physical exam or imaging. The VOTE classification system (Velum, Oropharynx, Tongue base, Epiglottis) is used to categorize a collapse as anteroposterior, lateral, or concentric [14].

DISE aids in preoperative planning by identifying the obstruction levels and gauging the response to maneuvers such as mandibular advancement. The mandibular pull-up (MPU) test can evaluate the improvement in airway patency during DISE [21].

Contraindications of DISE include a high ASA class, allergy to sedatives (e.g., propofol, midazolam), AHI > 70, and severe obesity. Key contraindications and practical considerations for DISE have been summarized in prior clinical reviews [20].

Reviewing the data on the failure rates of DISE-directed upper airway surgeries, the failure rates are quite low [22], and the collapsing patterns that are associated with failures (such as a complete circumferential collapse of the velum) can be predicted using DISE, thus confirming its value in customizing both single and multi-level surgical interventions [22,23].

#### 2.1.3. Clinical Assessment and Risk Stratification

A structured clinical assessment remains essential even when polysomnography (PSG) and drug-induced sleep endoscopy (DISE) are available because symptoms, comorbidities, and safety risks often drive prioritization and perioperative planning. A focused history should document snoring, witnessed apneas, nocturnal choking, insomnia, morning headaches, and daytime sleepiness, together with downstream consequences such as impaired concentration, mood changes, and occupational or motor-vehicle risk. Baseline cardiometabolic conditions (hypertension, arrhythmia, coronary disease, diabetes) and medication exposures (sedatives, opioids) should be recorded, because they influence anesthetic risk, postoperative monitoring needs, and the urgency of treatment.

Given diagnostic wait times in some publicly funded systems, screening tools can support triage and shared decision-making before definitive testing. The STOP and STOP-Bang questionnaires are simple, validated instruments that estimate the probability of OSA using symptoms and anthropometrics (including BMI and neck circumference) and can help identify patients who require expedited evaluation [24,25]. Symptom burden is best quantified with the Epworth Sleepiness Scale and complemented by partner-reported measures of snoring and witnessed apneas. Importantly, questionnaires do not replace PSG, but they help contextualize severity when PSG results and clinical impact do not align, and they offer a reproducible baseline for postoperative comparison and counseling [18].

Physical examination should emphasize modifiable contributors and anatomic targets. This includes nasal patency (septal deviation, turbinate hypertrophy), tonsil size, palate length and redundancy, tongue volume, craniofacial profile, and signs of mouth breathing. The Friedman staging framework and related oropharyngeal metrics remain useful for anticipating which patients are most likely to benefit from palatal-focused procedures, while BMI and neck circumference inform both technical feasibility and outcome expectations [2,26]. Clinicians should also address reversible aggravators (alcohol close to bedtime, untreated rhinitis, weight gain) and optimize adjunctive measures such as positional therapy or nasal treatment when appropriate. Evidence-based non-surgical alternatives, including mandibular advancement devices, should be discussed alongside CPAP and surgery, particularly in mild-to-moderate OSA or when CPAP is not tolerated [6,7,8].

From a protocol standpoint, defining CPAP intolerance or unwillingness explicitly is helpful. In practice, this includes documented inability to achieve adequate nightly use despite mask refitting, humidification, pressure adjustments, and behavioral support, or a clear preference for definitive anatomic intervention after informed counseling. The goal is not to position surgery as a substitute for every failed CPAP trial, but to provide a structured pathway for patients in whom CPAP is not a durable solution and for whom anatomic targets are identifiable on examination and DISE [18,20].

#### 2.1.4. Adjunctive Tests and Anatomical Phenotyping

Awake upper-airway evaluation complements PSG and DISE by documenting baseline anatomy and identifying pathology that may require treatment independent of OSA severity (e.g., severe nasal obstruction). Flexible nasoendoscopy in clinic allows assessment of the nasal cavity, nasopharynx, and laryngeal structures, and it helps rule out lesions or marked inflammatory disease that could confound surgical planning. This step also documents previous surgical changes and helps anticipate technical challenges, such as limited oral exposure or prominent gag reflex, that may influence the choice of technique and staging.

Imaging is not mandatory for all patients, but it can be valuable in selected situations: significant septal deformity, craniofacial skeletal discrepancies, suspected epiglottic pathology, or when prior surgery complicates anatomy. Cephalometry and computed tomography can describe skeletal relationships and airway volume and can support planning for maxillomandibular advancement (MMA) in patients with dentofacial abnormalities or severe disease [13,16]. However, static imaging cannot reliably capture dynamic collapse patterns; therefore, imaging should be interpreted as supportive rather than definitive for site-of-obstruction decisions. The protocol therefore treats DISE as the principal dynamic phenotyping tool, using VOTE-based characterization to guide targeted single- or multi-level intervention [20,27].

**Figure 1:**
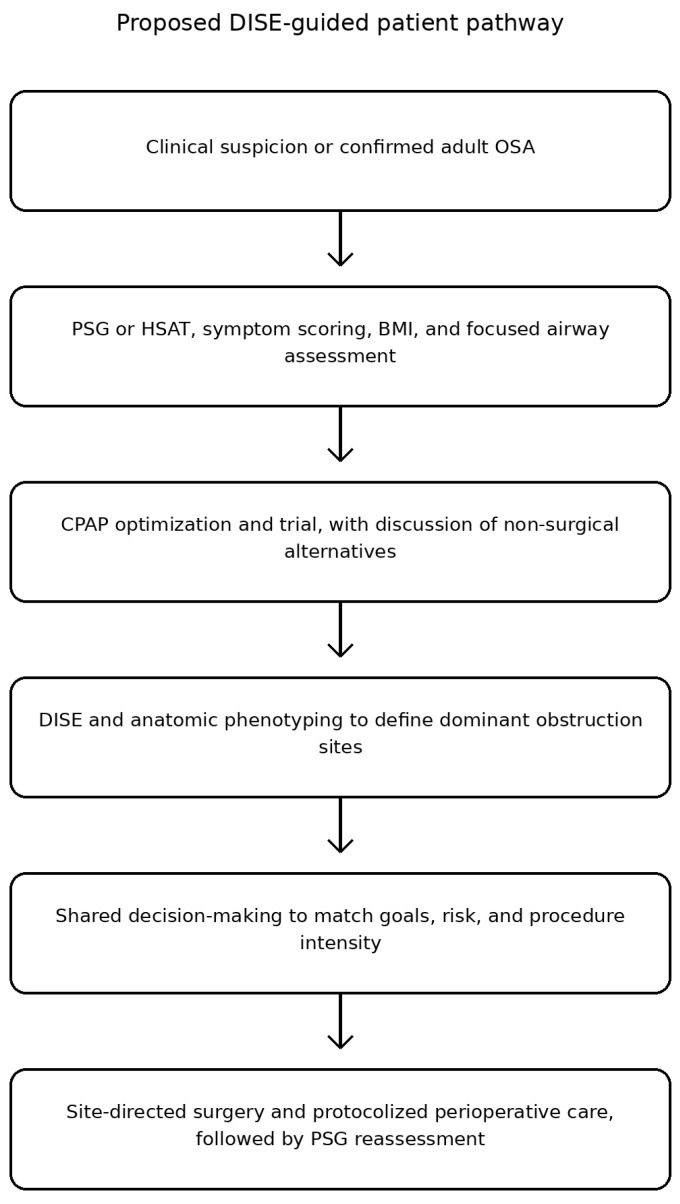
Patient pathway for the evaluation and management of obstructive sleep apnea in this protocol.

Finally, this protocol emphasizes outcome measures that extend beyond the apnea-hypopnea index (AHI). Patient-centered success incorporates symptoms, blood pressure, and hypoxemia burden in addition to PSG metrics, aligning with the SLEEP-GOAL framework that captures clinically meaningful improvement even when AHI changes are modest [19]. In practice, defining the intended outcome in advance (symptom relief, cardiometabolic risk reduction, CPAP independence, or procedural eligibility for upper airway stimulation) helps clinicians match the intensity of intervention to patient priorities and comorbidity profiles. The overall patient evaluation and management pathway used in this protocol is shown in Figure 1.

## 3. Surgical Protocol Overview

### 3.1. Indications for Surgery

Surgical intervention is considered in patients with CPAP intolerance or ineffectiveness, patients who do not want to use CPAP, patients with moderate-to-severe OSA confirmed on PSG, those with identifiable anatomical obstruction, and those with significant symptoms but a negative sleep study and elevated STOP-BANG or Epworth scores [18,24,25].

Before proceeding to surgery, patients should undergo a standardized optimization step to ensure that potentially reversible barriers to CPAP adherence have been addressed. This includes mask refitting, humidification, troubleshooting of leak and pressure intolerance, and education on realistic adaptation timelines. When CPAP remains unacceptable, the protocol supports a parallel discussion of alternatives such as mandibular advancement devices, positional therapy, and structured weight management, recognizing that combined therapy may be appropriate for some patients [6,7,8]. Patients should be counseled that surgical therapy aims to reduce anatomic collapsibility and improve symptoms and health outcomes, but it may not eliminate the need for adjunctive therapy in every case, particularly in severe disease or in those with persistent obesity [2].

Patient selection should also incorporate perioperative risk and long-term goals. Individuals with significant cardiopulmonary comorbidity, high anesthetic risk, or extremely severe OSA (e.g., AHI above the thresholds used to exclude DISE candidates) may require staged procedures, planned postoperative monitoring, or referral to tertiary centers with multidisciplinary support [20]. In eligible patients, upper airway stimulation (UAS) can be considered as an alternative to extensive soft-tissue surgery; however, complete concentric collapse of the velum on DISE remains a disqualifying finding unless corrected with palatal pharyngoplasty [23,28]. Setting these decision points explicitly within the protocol helps align intervention intensity with patient preference, resource availability, and expected benefit.

### 3.2. Multi-Level Surgical Approach

Due to the multi-level nature of OSA, customized surgeries depending upon findings from DISE are mandatory [13,14]. Most common procedures are as follows: Uvulopalatopharyngoplasty (UPPP) remains the most common surgical procedure performed for the treatment of sleep apnea worldwide. Many surgeons specializing in OSA are avoiding more ablative techniques of UPPP that include removal of the uvula. This trend is particularly so for laser-assisted UPPP, which, according to a meta-analysis, worsens AHI in 44% of patients. Isolated soft palate surgeries have the highest success rates in patients with Friedman stage I [26]; however, in practice, various UPPP methods are more commonly used in multi-level surgical procedures to enhance success rates [29]. An isolated UPPP may still be required as a part of a staged approach for upper airway stimulation (UAS). The observation of complete concentric collapse (CCC) of the velum during DISE remains a disqualifying criterion for UAS; however, this collapse pattern can be corrected with palatal pharyngoplasty and restore UAS eligibility [23,30].

The sequential continuum of obstructive sleep apnea (OSA) and untreated obstructive sleep apnea (OSA) in retrolingual areas are recognized major contributors to surgical failures. Depending on the surgeon’s discretion, the removal of lingual tonsils and subcutaneous fat (adipose) tissue at the tongue base may be completed via coblation-, laser-, or robotic-assisted surgical techniques. The removal of tissue in this area of the tongue-base region of the retrolingual should be complemented in surgical techniques by fixing the epiglottis to the tongue base to prevent epiglottic inversion (collapse). Palatoplasty (surgical removal of the soft palate) is also indicated when drug-induced sleep endoscopy (DISE) demonstrates involvement of the epiglottis [13,31,32].

The procedure involves osteotomies of the maxilla and mandible, which are then advanced, typically with counterclockwise rotation. This creates more space for intraoral soft tissues and enhances the positioning of the upper airway dilator muscles. The general criteria for considering MMA are as follows: (1) moderate-to-severe OSA with or without previous phase I surgery, (2) OSA of any severity with considerable dentofacial abnormalities, and (3) the presence of concentric or lateral pharyngeal wall collapse on deep inspiration sleep endoscopy (DISE) [13].

Single-stage surgery may suffice for selected patients, while staged interventions may be preferable for severe or comorbid cases [13,31].

### 3.3. Perioperative Management

#### 3.3.1. Preoperative Evaluation

**Figure 2:**
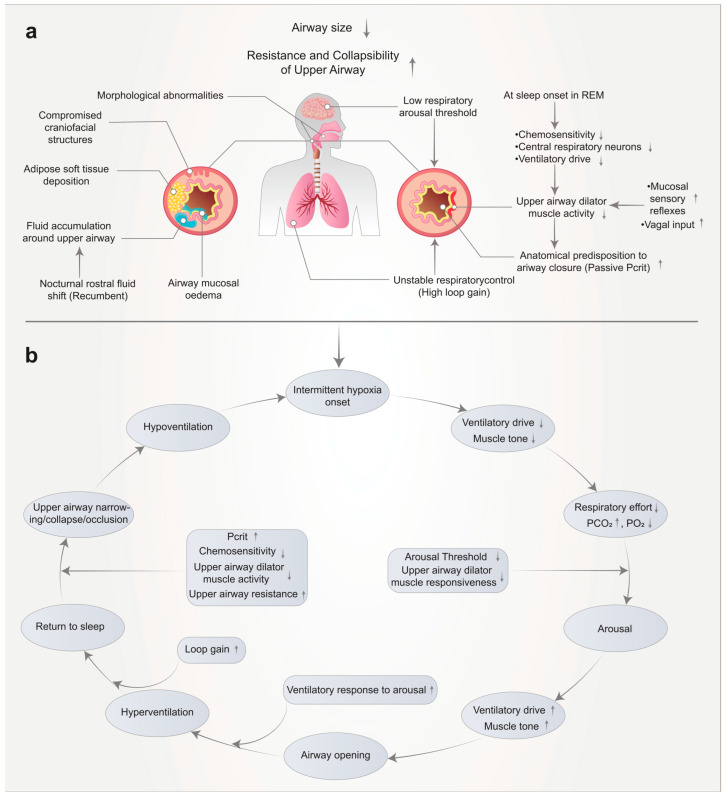
Mechanisms influencing upper airway collapse in obstructive sleep apnea syndrome (OSAS) and interplay between contributing factors (**a**,**b**). Reproduced from Lv et al. under CC BY 4.0 [1].

BMI and OSA: most individuals diagnosed with sleep apnea are overweight. There is a robust and consistent link between the severity of the condition and obesity [2]. Based on our understanding of the underlying mechanisms and the influence of fat accumulation around the upper airway, this relationship is causal in nature. Weight loss not only alleviates sleep apnea but can also lead to its complete resolution. Therefore, for many patients, sleep apnea serves as an indicator of obesity, suggesting that addressing the root cause is preferable to merely treating the symptoms. This perspective is especially relevant from a public health standpoint, as the connection between obesity and cardiovascular disease is strong and well-documented. By managing obesity, we can not only mitigate sleep apnea but also decrease the likelihood of heart disease and strokes. This strategy has the potential to enhance the overall health of both individuals and the community [2,4]. Figure 2 summarizes the multifactorial pathophysiology of obstructive sleep apnea syndrome (OSAS). Panel (a) illustrates the main anatomical and non-anatomical factors that reduce upper airway size and increase its resistance and collapsibility, including craniofacial abnormalities, adipose soft tissue deposition, mucosal edema, fluid shift, low respiratory arousal threshold, unstable ventilatory control, and reduced upper airway dilator muscle activity during sleep. Panel (b) depicts the cyclic sequence of obstructive events, in which upper airway narrowing or collapse leads to hypoventilation and intermittent hypoxia, followed by changes in ventilatory drive, respiratory effort, and arousal, with temporary airway reopening and recurrence of the cycle after return to sleep [1].

#### 3.3.2. Intraoperative Protocol

The surgical procedure involves a reverse OR table setup to enhance the airway exposure, followed by a sequential approach consisting of a tonsillectomy, UPPP, and tongue base reduction. Coblation ensures precision during the procedure. Laryngoscope access is facilitated using a Lindholm or laryngoscope (KARL STORZ Endoskope, Tuttlingen, Germany). Hemostasis and analgesia are achieved with local bupivacaine and epinephrine, specifically 0.5% Marcaine with 1:100,000 or 1:200,000 epinephrine.

#### 3.3.3. Postoperative Care

The patient should be discharged with pain medication such as Celecoxib 200 mg oral capsule (liquid) 200 mg 1 cap BID, along with Tylenol 650 mg Q8H, for 7 days. We also recommend Magic mouth wash, which contains an aluminum–magnesium hydroxide suspension, diphenhydramine 2.5 mg\mL LIQ, and lidocaine viscous 2%. Monitoring for airway compromise and hemorrhage is mandatory, especially in high-risk patients. If the patient is diagnosed with severe OSA, a PSG at 6 months post operation is recommended, where one can consider adjunctive therapies post operation (e.g., positional therapy, oral appliances) [18,33,34].

Postoperative disposition should be individualized. Patients with severe OSA, significant cardiopulmonary disease, difficult airway features, or extensive multi-level intervention may require overnight monitoring for airway compromise, hypoxemia, and hemorrhage. Conversely, carefully selected patients can be managed safely in ambulatory settings when standardized protocols for analgesia, hydration, and emergency access are in place [35]. In all settings, clinicians should provide clear instructions regarding warning signs (bleeding, progressive dyspnea, inability to tolerate oral intake), hydration goals, and graded return to diet and activity.

Follow-up should be protocolized rather than ad hoc. Early review (within 1–2 weeks) focuses on pain control, wound assessment, and hydration, while later visits address symptom response, snoring persistence, and adherence to adjunctive measures. Objective reassessment with PSG is typically performed after healing, commonly at 3–6 months, with timing tailored to disease severity and the extent of surgery [18]. Outcome assessment should incorporate both PSG metrics and patient-centered measures (Epworth score, blood pressure, hypoxemia load) in line with SLEEP-GOAL principles, and residual disease should trigger a structured step-up plan (oral appliance therapy, positional therapy, or consideration of additional site-directed surgery) rather than an unstructured return to CPAP alone [6,7,8,19].

## 4. Discussion

Obstructive sleep apnea (OSA) treatment has progressed from single soft-palate procedures to multi-level surgeries based on distinct phenotype and DISE findings [13,31,32,36]. This change indicates that the complex issues of OSA are multi-dimensional and each OSA patient may benefit from a tailored combination of nasal, palatine, tongue-base, and skeletal surgeries.

The protocol provided in the article standardizes the connection of pre-operative assessments, DISE-based classifications, and tiered surgical options into a single pathway. This pathway standardization contributes to the contemporary focus on defining surgical success beyond apnea hypopnea indices (AHI) to include patient-centered functionality, symptoms, cardiometabolic complications, and the overall success of the system [21]. The preference of Saudi Arabia and Canada as the primary exemplars of the same principles being implemented in disparate health systems is not meant to preclude the imposition of the protocol to other health systems [31].

From an implementation perspective, the value of a protocol is amplified in health systems where access to sleep testing and specialist care is constrained. In Canada, published assessments of wait times highlight how delays in diagnostic pathways can extend many months and contribute to undertreatment [11,12]. In Saudi Arabia, sleep-medicine service availability and awareness vary by region, and standardized surgical pathways may be underutilized despite a high burden of disease [10]. A pragmatic, DISE-guided algorithm can therefore function not only as a technical guide for operative planning, but also as a common language for multidisciplinary decision-making across ENT, sleep medicine, anesthesia, dentistry, and weight-management services. Embedding this pathway within routine documentation (structured DISE reporting, standardized outcome tracking, and predefined criteria for outpatient versus inpatient management) supports safety and reproducibility, and it facilitates audit and quality improvement as recommended in contemporary surgical care models [20,35].

### 4.1. Limitations

A major portion of this work is based almost exclusively on existing literature and the anecdotal accounts of high-volume sleep surgeons. It does not include a prospective cohort treated stringently with the proposed algorithm. Most of the cited resources, studies, and literature come from expert and specialized centers. This “expert” literature sourcing may lead to further inaccessibility and lack of generalizability to resource-scarce hospitals, unimaginable DISE resources, and alien patient demographics. Moreover, the literature describing long term-weight management agents, hypoglossal nerve stimulation, and even complex multi-level combinations in patients with high negative BMI is sparse.

### 4.2. Future Recommendations

Building on the above, research must seek to obtain future studies that are mostly prospective and aim to validate this guide across heterogeneous centers, with the aim of standardized reporting on SLEEP-GOAL and other multi-dimensional outcome measures in addition to AHI. We are in need of studies on comparative effectiveness and cost-effectiveness for determining the timing of the greatest benefit of the various modalities, i.e., surgery, CPAP, oral appliances, weight-loss, and combinations, in varying clinical and healthcare system contexts. In areas with low sleep lab and DISE availability, the use of the protocol, even with the addition of telemedicine, stepwise introduction of more complex procedures, and adjustments to the pathway, will be important to demonstrate that protocol-driven care extends beyond the Canadian and Saudi frameworks [31].

## 5. Conclusions

Obstructive sleep apnea entails a number of comorbidities that require a multidisciplinary systematic approach rather than singular interventions. For adult patients that are CPAP-intolerant and/or uncooperative, integrating empirical assessments, DISE-informed multilevel sleep surgery and structured perioperative pathways is a rational approach to optimize symptom relief and minimize cardiometabolic complications.

We intentionally designed this protocol to be agnostic to geography and health systems. As such, we draw on examples from Saudi Arabia and Canada to illustrate how systematic phenotyping, collaborative team decision-making, and progressive surgical complexity can be operationalized in disparate systems and organizational levels. With the necessary adaptations, we argue that the growing utilization of such protocol-based approaches, along with outcome assessments and iterative modifications, can achieve greater levels of consistency in care and enhance the outcomes of patients with OSA on a global level.

## Data Availability

The original contributions presented in this study are included in the article. Further inquiries can be directed to the corresponding author.
